# Intermittent Tacrolimus Treatment Delays CD8+ Tumor‐Infiltrating Lymphocyte Exhaustion and Enhances PD1 Blockade Therapy in Melanoma‐Bearing Mice

**DOI:** 10.1155/jimr/8444562

**Published:** 2026-02-16

**Authors:** Darong Chen, Sisi Feng, Zhaolong Chen, Zhisheng Weng, Shu Zhang, Junjin Lin, Zili Wang

**Affiliations:** ^1^ Department of Neurosurgery, Neurosurgery Research Institute, The First Affiliated Hospital, Fujian Medical University, Fuzhou, 350004, Fujian, China, fjmu.edu.cn; ^2^ Department of Neurosurgery, National Regional Medical Center, Binhai Campus of the First Affiliated Hospital, Fujian Medical University, Fuzhou, 350026, Fujian, China, fjmu.edu.cn; ^3^ Fujian Provincial Institutes of Brain Disorders and Brain Sciences, The First Affiliated Hospital, Fujian Medical University, Fuzhou, 350004, Fujian, China, fjmu.edu.cn; ^4^ Fujian Provincial Key Laboratory of Precision Medicine for Cancer, The First Affiliated Hospital, Fujian Medical University, Fuzhou, 350004, Fujian, China, fjmu.edu.cn; ^5^ Department of Pathology, The First Affiliated Hospital, Fujian Medical University, Fuzhou, 350004, Fujian, China, fjmu.edu.cn; ^6^ Department of Pathology, National Regional Medical Center, Binhai Campus of the First Affiliated Hospital, Fujian Medical University, Fuzhou, 350026, Fujian, China, fjmu.edu.cn; ^7^ Department of Internal Medicine, Luoyuan County General Hospital, Fuzhou, 350600, Fujian, China; ^8^ Department of Oncology, The First Affiliated Hospital, Fujian Medical University, Fuzhou, 350004, Fujian, China, fjmu.edu.cn; ^9^ Department of Oncology, National Regional Medical Center, Binhai Campus of the First Affiliated Hospital, Fujian Medical University, Fuzhou, 350026, Fujian, China, fjmu.edu.cn; ^10^ Public Technology Service Center, Fujian Medical University, Fuzhou, 350122, Fujian, China, fjmu.edu.cn

**Keywords:** over-activation, PD1 blockade, T cell exhaustion, tacrolimu

## Abstract

Continuous antigen exposure initiates the developmental process of CD8+ T cell exhaustion, accompanied by a gradual increase in the expression of inhibitory receptors (IRs). Our previous study conceptualized T cell exhaustion as an over‐activation status induced by chronic antigen stimuli. To intervene in the developmental process of CD8+ T cell exhaustion, we adjusted the over‐activation status by intermittently blocking T cell activation signals with tacrolimus, a T cell activation inhibitor. Melanoma‐bearing mice received intermittent tacrolimus (IT) and/or programmed cell death 1 (PD1) blockade antibody. Exhaustion phenotype of CD8+ tumor‐infiltrating lymphocytes (CD8+TILs) was detected by flow cytometry. The expression levels of IRs (PD1 and CD223), as well as memory markers (Ly108 and CD127), were utilized to demarcate the extent of CD8+TILs exhaustion. Then, tyrosinase‐related protein 2 peptide (Trp2_180–188_) was used to detect tumor‐specific CD8+ TILs. Furthermore, tumor growth rate and tumor weight were also evaluated. We found that IT and/or PD1 blockade enhanced the infiltration of functional CD8+TILs. The findings demonstrated that both IT therapy and PD1 blockade enhanced the antitumor immunity. Combining IT and PD1 blockade led to a greater reduction in tumor growth rate and tumor weight. IT therapy also improved the response of CD8+TILs to melanoma‐specific Trp2_180–188_ peptide. A detailed analysis of the CD8+TILs showed that Ly108 and CD127 reduced dramatically as the expression of PD1 increased. In comparison to PD1 blockade, combined treatment (IT plus PD1 blockade) significantly increased the number of intermediate IRs‐expressing CD8+TILs, while reducing the number of high IRs‐expressing CD8+TILs. These results indicated that combined treatment enhanced the function and decelerated the increase in IRs expression of CD8+TILs, deferring the developmental process of CD8+TILs exhaustion.

## 1. Introduction

In the context of chronic infections and emergence of tumors, effector T cells exhibit a remarkable failure to differentiate into a memory phenotype. This failure is characterized by a progressive loss in effector functions, eventually leading to the deletion of the responsive cells [1, 2]. T cell factor family member TCF1 (*Tcf7*) is a key transcription factor essential for memory cell generation and self‐renewal [3, 4]. Similar to TCF1, CD127 is also associated with memory T cells [5]. Both are crucial for maintaining self‐renewal and are silenced during exhaustion differentiation [6]. Exhausted CD8+ T cells (Tex) exhibit low levels of memory markers, like CD127 and TCF1, but high levels of inhibitory receptors (IRs), such as programmed cell death 1 (PD1) receptor, cytotoxic T lymphocyte‐associated antigen 4 (CTLA4), lymphocyte activation gene‐3 (LAG3, known as CD223), T cell immunoglobulin domain, and mucin domain‐3 (TIM3) [1, 7, 8].

The progression of exhaustion unfolds in a hierarchical manner, with the initial loss of effector functions, including diminished IL‐2 production and reduced proliferative capacity. Subsequently, the modified expression of crucial transcription factors triggers the persistent upregulation of various IRs, such as PD1 and CD223. Recent studies have identified the developmental framework of Tex: Progenitor Tex subsets gradually lost TCF1 and CD127, while gaining PD1 and CD223 as it divided and converted to intermediate and terminally exhausted subsets [1, 9]. The study has shown the expression of TCF1 can be surrogated by Ly108 (also known as Slamf6) in Tex. Progenitor Tex expressed high levels of both TCF1 and Ly108, whereas very few terminally Tex expressed TCF1 or Ly108 [9, 10]. Therefore, PD1, CD223, CD127, and Ly108 can serve as discerning markers capable of distinguishing between the early and late stages of exhaustion [9, 11].

Tex can be attributed to several factors, such as regulatory T cells, inhibitory signals from cytokines, and cell surface IRs, among others. However, a key characteristic appears to be persistent antigen exposure rather than acute termination or intermittent exposure. The degree of exhaustion is correlated with the intensity and duration of stimulation received by the T cell receptor (TCR). Higher antigen loads, larger amounts of epitopes, and prolonged exposure can all lead to more severe exhaustion [8, 12]. Therefore, in the previous study, the authors regarded T cell exhaustion as an over‐activation status induced by chronic antigen stimulation and hypothesized that intermittent blockade of the TCR signal to regulate over‐activation signals could slow down the development of T cell exhaustion [13].

We noticed that T cell exhaustion can be ameliorated by providing co‐stimulatory signals, such as CD2, 4‐1BB, and CD40L [1, 14, 15]. This evidence suggests that Tex may be caused by lack of co‐stimulatory signals, other than over‐activation by prolonged antigen stimuli. A possible explanation for this puzzle would be an imbalance between TCR signal and co‐stimulatory signal during chronic antigen exposure. We know that two proper signals are needed to activate naïve CD8+ T cells: first the TCR signal (antigen stimulation) and second the co‐stimulatory signal, for example, 4‐1BB and CD28. In the case of prolonged antigen stimulation, the TCR signal might override the co‐stimulatory signal, resulting in impaired effector function. In this case, we can provide stronger co‐stimulatory signals to rebalance two signals. Major evidence for the imbalance of two signals is that CD28/B7 costimulatory pathway is essential for effective PD1/CTLA4 therapy [14, 16, 17]. On the other hand, it is also rationale to adjust the relatively stronger TCR signal to a proper extent to rematch co‐stimulatory signals.

Tacrolimus is a calcineurin inhibitor that suppresses calcium‐dependent phosphatase, hindering the dephosphorylation required for the nuclear translocation of the transcription factor family (nuclear factor of activated T‐cells [NFAT]), thereby impeding T cell activation. In the earlier study of the authors, intermittent blockade of TCR signals with tacrolimus facilitated the infiltration of functional CD8+ tumor‐infiltrating lymphocytes (CD8+TILs) in melanoma‐bearing mice [13].

The current study aimed to further compare PD1 blockade therapy with intermittent tacrolimus (IT) treatment in melanoma‐bearing mice. Specifically, it aimed to achieve the following specific objectives: ① to assess the impact of IT and/or PD1 blockade on the enhancement of functional CD8+TILs, ② to investigate the potential of IT in mitigating the exhaustion of CD8+TILs, ③ to examine the influence of IT and/or PD1 blockade on the memory markers of CD8+TILs, and ④ to explore the synergistic antitumor effects of IT and PD1 blockade in melanoma‐bearing mice.

## 2. Materials and Methods

### 2.1. Cell Culture and Mice

Mouse B16F10 melanoma cell line (RRID: CVCL_0159) was obtained from the Cell Bank of the Chinese Academy of Sciences (Shanghai, China). These cells are characterized by the production of melanin [13, 18]. All experiments were performed with mycoplasma‐free cells. Cells were cultured in DMEM medium supplemented with 10% FBS and maintained in a humidified incubator containing 5% CO_2_ at 37°C.

Pathogen‐free 8–10‐week‐old C57BL/6 female mice (RRID: MGI:2159769) were purchased from Shanghai SLAC Laboratory Animal Co., Ltd. Animal care and experiments were conducted following the guidelines and policies of the Animal Care and Use Committee of Fujian Medical University, China (IACUC FJMU 2024‐Y‐2438).

### 2.2. Reagents and Antibodies

Tacrolimus (Sigma, F4679) was dissolved in DMSO at a concentration of 20 mg/mL immediately after receiving and stored at −20°C. For in vivo administration, the dissolved tacrolimus was first diluted by PBS to 5 mg/mL, then diluted again by PBS to 1 mg/mL. After that, tacrolimus solution was dilued by a third round of PBS to reach a final concentration of 0.2 mg/mL (the percentage of DMSO is 1%). Essential reagents such as 7‐Amino‐actinomycin D (7‐AAD), APC‐Cy7‐conjugated anti‐mouse CD4 (BD, 552,051), BUV395‐conjugated anti‐mouse CD45 (BD, 564,279), BV510‐conjugated anti‐mouse CD8 (BD, 563,068), APC‐conjugated anti‐mouse CD3 (BD, 565,643), PE‐conjugated anti‐mouse PD1 (Elabscience, E‐AB‐F1131D), BV711‐conjugated anti‐mouse CD223 (BD, 563,179), BV421‐conjugated anti‐mouse CD107a (BD, 564,347), BV650‐conjugated anti‐mouse Tim3 (BD, 757,623), FITC‐conjugated anti‐mouse CD127 (eBioscience, 11‐1271‐82), BUV496‐conjugated Ly108 (BD, 750,046), PE‐conjugated IFN‐γ (Invitrogen, 12‐7311‐82), BV421‐conjugated TNF‐α (BD, 563,387), and PE‐Cy7‐conjugated IL‐2 (BD, 560,538) were used for flow cytometry testing. The FACS buffer composition comprised PBS with 3% fetal bovine serum.

### 2.3. Antitumor Effects

C57BL/6 mice received subcutaneous injections of 1 × 10^6^ B16F10 cells in a shaved rear flank. Subcutaneous injections were performed after anesthesia with isoflurane inhalation. After 6–9 days, when tumors became palpable, the mice were randomly assigned to the following treatment groups: vehicle (V) group, IT treatment group, PD1 blockade (P) group, and the combined treatment group (IT + PD1 blockade, ITP). Stratified randomization (based on tumor size) was used to allocate mice to groups. Tacrolimus was administered intraperitoneally (i.p.) at a dose of 2.25 mg/kg every 5 days. The V group received an equivalent volume of the vehicle (PBS + 1% DMSO) via i.p. injection concurrently with tacrolimus administration. PD1 blocking antibody was i.p. administered at a dose of 200 μg 1 day before tacrolimus treatment. When PD1 blocking antibody was administered, mice in the V or IT groups received 200 μg of isotype control antibodies. Tumor volume was assessed every 2–3 days by measuring two opposing diameters (length = L, width = W). Mice were euthanized when the tumor volume reached or exceeded 600 mm^3^ (volume = V, *V* = L × W × W/2). Mice were euthanized by carbon dioxide asphyxiation. Tumor measurement was performed blinded to treatment groups.

### 2.4. Flow Cytometry

For flow cytometry analysis, tumors were surgically excised for analysis 4 days post the first dose of tacrolimus. Then, the tumors were cut into 2‐mm pieces and incubated in complete RPMI 1640, comprising 10% FBS, collagenase A (2 mg/mL), and DNase I (1 mg/mL), at 37°C for 15 min. Subsequently, the tumor tissues were dissociated using the gentleMACS Dissociator (Miltenyi Biotec). Upon obtaining single‐cell suspensions, these were passed through a 70‐μm cell strainer and washed once in complete RPMI 1640. The tumor cell suspensions underwent lysis with red blood cell lysing buffer for 3 min on ice. Following this, the tumor cells were resuspended in FACS buffer and blocked with mouse IgG mAb. Surface antibodies, including CD45, CD3, CD8, CD4, PD1, CD223, CD107a, Ly108, and CD127, were applied to stain tumor tissue single‐cell suspensions for 30 min. A viability dye (7‐AAD) was used for all surface panels. In some experiments, intracellular staining (ICS) was performed as previously described [13]. Briefly, tumor single‐cell suspensions were stimulated with PMA (final concentration: 50 ng/mL) and ionomycin (final concentration: 1 µg/mL) in the presence of GolgiPlug (1 µL/mL, BD Biosciences, 555,029) for 4 h at 37°C. In order to detect tumor specific CD8+TILs, tyrosinase‐related protein 2 peptide (Trp2_180–188,_ SVYDFFVWL) was added to the tumor cell suspension at 10 μg/mL. After stimulation for 4 h, the cells were first stained with Fixable Viability Stain 575V (BD, 565,694) to identify alive cells. Then, the cells were stained with surface antibodies. After that, ICS was performed using the Fixation/Permeabilization kit (BD, 554,714) according to the manufacturer’s instructions. Afterward, the cells were washed twice and stained with anti‐mouse IFN‐γ, anti‐mouse TNF‐α, and anti‐mouse IL‐2 for 30 min on ice. At last, the cells were washed by FACS buffer. Data were acquired on a BD LSRFortessa X‐20 and analyzed with FlowJo v10.x; at least 3000 live CD8+TILs were collected per sample. We confirmed that FMO and compensation controls were included for each panel.

### 2.5. Statistical Analysis

Results are presented as the mean ± SEM. Tumor growth curves were analyzed by repeated‐measures two‐way ANOVA with Greenhouse–Geisser correction and Tukey’s post hoc test. Ly108 and CD127 expression levels among different cell populations of each sample were compared using paired *t*‐test. Additional differences between groups were evaluated using unpaired Student’s *t*‐test. Data analyses and graphical representations were conducted using Prism 8.0.1 software (GraphPad). A significance level of *p* < 0.05 was considered statistically significant. All tests are two‐tailed. The robustness of the experimental data was confirmed through replication in at least three independent experiments. Exact *p*‐values are reported in each figure. Sample sizes (*n*) represented biological replicates (individual mice) and were indicated in each figure legend.

## 3. Results

### 3.1. PD1 and CD223 Defined Intermediate and Terminally Exhausted Subsets of CD8+TILs in a Mice Melanoma Model

The developmental trajectory of Tex cells has been previously documented, indicating that progenitor exhausted subsets progressively increase the expression of IRs and downregulate the expression of memory markers as they divide, ultimately transforming into a terminally exhausted subset, where two Ly108+ progenitor subsets, a third intermediate (Ly108−) and a fourth terminally exhausted (Ly108−) subsets, were identified [9, 10]. Compared with the intermediate subset, the terminally exhausted subset upregulated the proteins for IRs (PD1, CD223, and Tim3) [9, 11]. Consequently, this study assessed PD1, CD223, and Tim3 expression on CD8+TILs of melanoma‐bearing mice. The flow cytometry gating strategies are illustrated in Figure S1. As shown in Figure 1, when gating on CD8+TILs, the PD1+CD223+ population exhibited significantly higher mean fluorescence intensity (MFI) of PD1, CD223, and Tim3 compared to the PD1+CD223− population (Figure 1A,B). Therefore, PD1+CD223+CD8+TILs were classified as high PD1 expressing CD8+TILs (PD1hi CD8+TILs), while PD1+CD223‐CD8+TILs were considered as intermediate PD1 expressing CD8+TILs (PD1int CD8+TILs). The expression of Tim3 was similar to that of CD223. PD1hi population exhibited highest expression level of both Tim3 and CD223.

Figure 1The expression of PD1, CD223, and Tim3 in CD8+TILs (*n* = 5). (A) Upper panel: Gating on CD8+TILs, contour plots showed the PD1, CD223, and Tim3 expression in CD8+TILs from melanoma‐bearing mice. Lower panel: Histograms showed the MFI of PD1, CD223, and Tim3 between PD1hi and PD1int CD8+TILs from A (dashed red: PD1int subset; solid blue: PD1hi subset). (B) Differences between PD1int and PD1hi populations were evaluated using an unpaired Student’s t‐test. The experimental data were confirmed through replication in five independent experiments. MFI, mean fluorescence intensity; PD1hi, high PD1 expressing subset (PD1+CD223+); PD1int, intermediate PD1 expressing subset (PD1+CD223−); TILs, tumor‐infiltrating lymphocytes.

**Figure:**
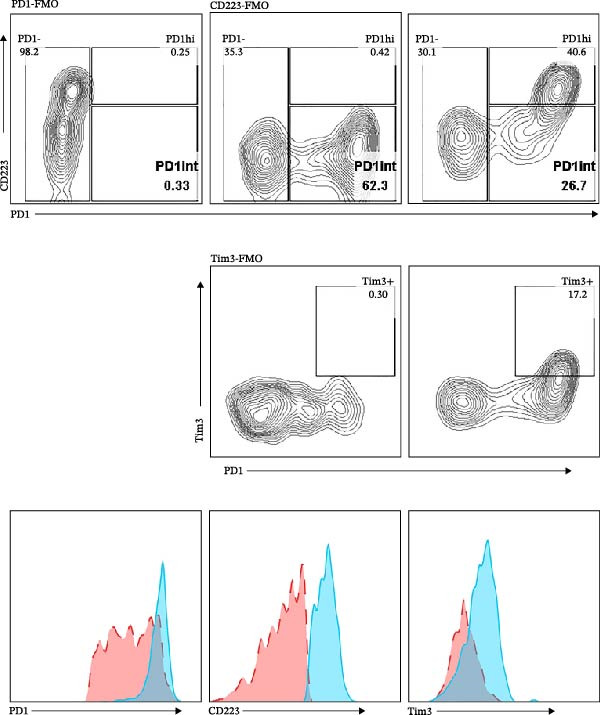
(A)

**Figure:**
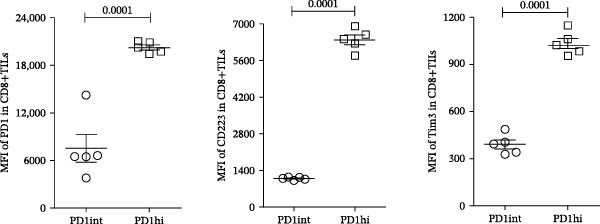
(B)

We further evaluated the memory markers (CD127 and Ly108, a surrogate for TCF1) and functional marker CD107a in PD1hi, PD1int, and PD1 negative (PD1−) populations of CD8+TILs. Compared to PD1‐ population, the expression of Ly108 and CD127 on PD1+ populations (PD1hi and PD1int) reduced dramatically (Figure 2A,C,D). Nearly no cells expressed Ly108 or CD127 in the PD1hi population of CD8+TILs (Figure 2C,D). The expression of CD107a correlated positively with PD1. The average percentage of CD107a+ cells in PD1‐, PD1int, and PD1hi populations was 12%, 34%, and 43% (Figure 2B,E).

Figure 2The expression of Ly108, CD127, and CD107a in PD1hi, PD1int, and PD1 negative populations of CD8+TILs (*n* = 5). (A) Gating on different populations of CD8+TILs, contour plots showed the expression of Ly108 and CD127. (B) Gating on different populations of CD8+TILs, contour plots showed the expression of CD107a. (C–E) Frequencies of Ly108+, CD127+, and CD107a+ cells among indicated CD8+TILs populations. (C) The ratio of Ly108+ cells in PD1‐, PD1int, and PD1hi CD8+TILs. (D) The ratio of CD127+ cells in PD1‐, PD1int, and PD1hi CD8+TILs. (E) The ratio of CD107a+ cells in PD1−, PD1int and PD1hi CD8+TILs. Ly108, CD107a, and CD127 expression levels among different cell populations of each sample were compared using a paired t‐test. The experimental data were confirmed through replication in three independent experiments. PD1hi, high PD1 expressing subset (PD1+CD223+); PD1int, intermediate PD1 expressing subset (PD1+CD223−); PD1‐, PD1 negative subset; TILs, tumor‐infiltrating lymphocytes.

**Figure:**
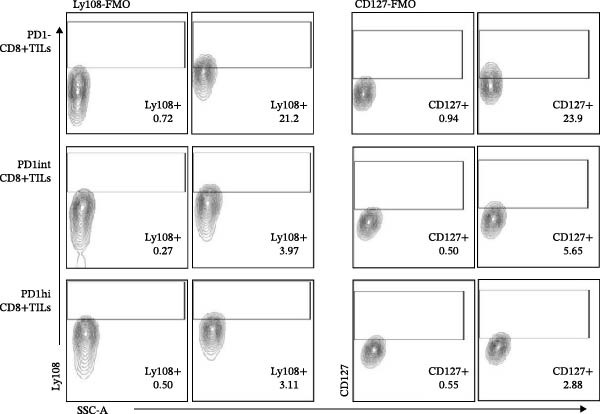
(A)

**Figure:**
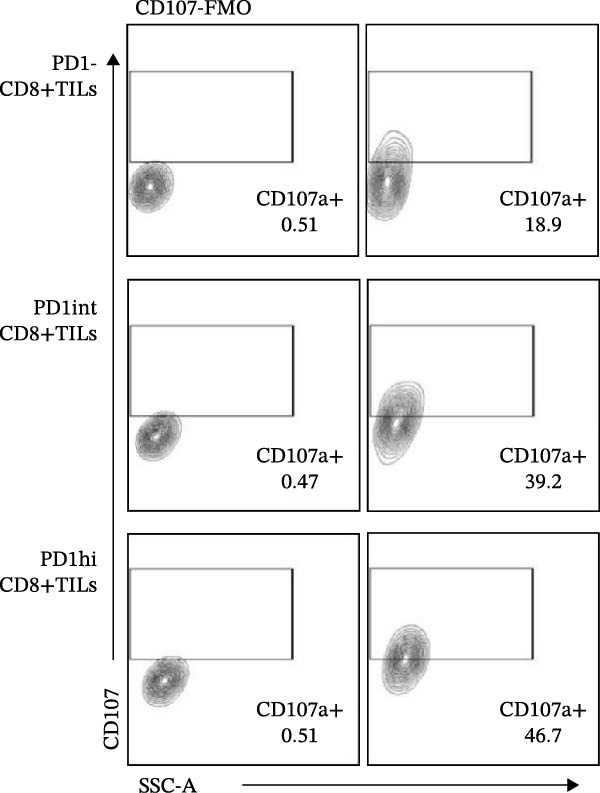
(B)

**Figure:**
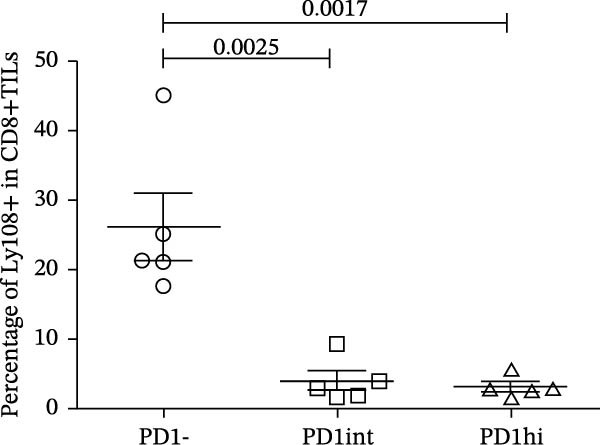
(C)

**Figure:**
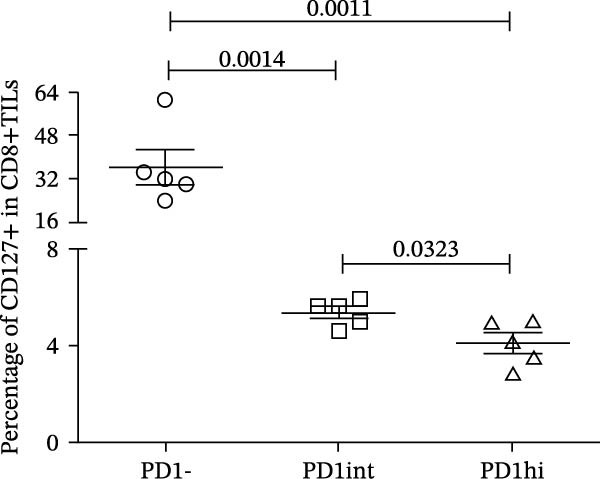
(D)

**Figure:**
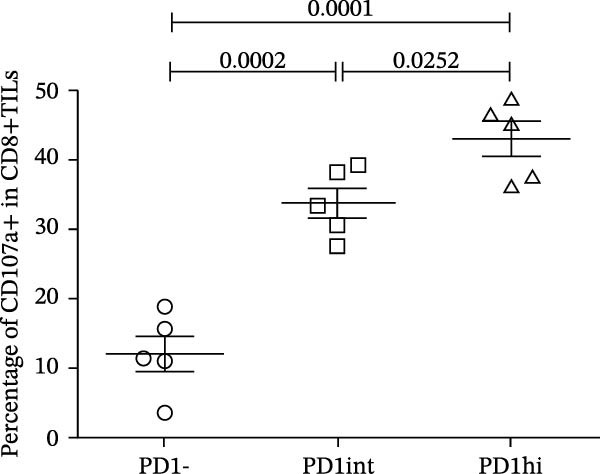
(E)

Figures 1 and 2 showed the profiles of CD8+TILs in melanoma‐bearing mice, including multiple IRs and memory markers. The results demonstrated that the phenotype of PD1int CD8+TILs in our study was similar to the intermediate Tex reported previously; both were Ly108 negative with low expression of IRs [9]. The phenotype of PD1hi CD8+TILs was similar to the terminally exhausted subset reported previously; both were Ly108 negative but expressed high levels of IRs [9].

### 3.2. IT and/or PD1 Blockade Increased the Number of Functional CD8+TILs

This study aimed to assess the quantity of functional CD8+TILs posttreatment. Functional marker, CD107a, was found in the membrane of cytotoxic granules and transiently expressed on the cell surface upon granule release. Surface CD107a staining has been proven to mirror the profiles of production of cytokines (such as IFN‐γ and TNF‐α) and granzyme B [9, 19, 20].

As illustrated in Figure 3A–C, the PD1 blockade group and the combined treatment group exhibited a significant increase in the number of CD8+TILs. Notably, tacrolimus did not impede the infiltration of CD8+TILs compared to the V group; instead, it augmented the proportion of functional CD8+TILs (CD107a+CD8+TILs, Figure 3A–D). This percentage closely approximated that observed in the PD1 blockade group (Figure 3D). These findings underscored the capacity of IT to enhance the functionality of CD8+TILs.

Figure 3The number of functional CD8+TILs (*n* = 11 or 12 in each group). (A) Upper panel: gating on CD3+TILs, contour plots showed the CD8+TILs. Lower panel: gating on CD8+TILs, contour plots showed the CD107a+ cells. (B) The ratio of CD3+TILs in CD45+ immune cells. (C) The ratio of CD8+TILs in CD3+TILs. (D) The ratio of CD107a+ cells in CD8+TILs. Differences among different treatment groups were evaluated using an unpaired Student’s *t*‐test. The experimental data were confirmed through replication in three independent experiments. IT, intermittent tacrolimus treatment group; ITP, combined treatment group (intermittent tacrolimus + PD1 blockade); P, PD1 blockade group; TILs, tumor‐infiltrating lymphocytes; V, vehicle group.

**Figure:**
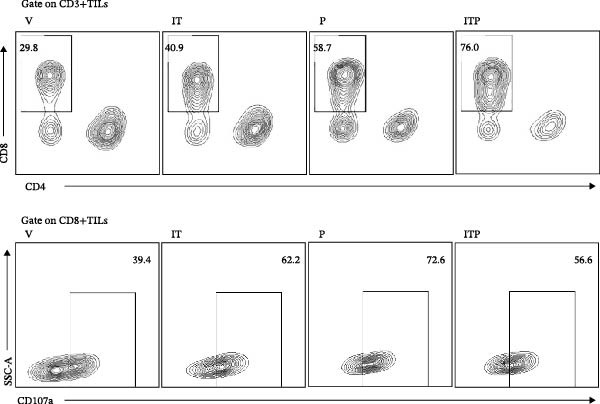
(A)

**Figure:**
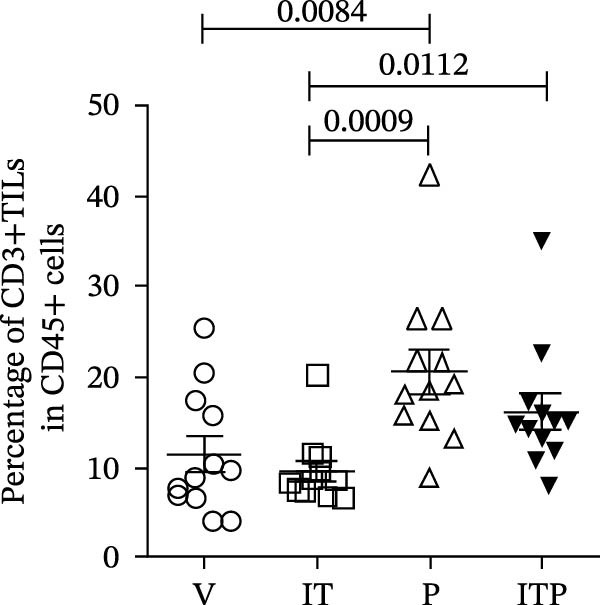
(B)

**Figure:**
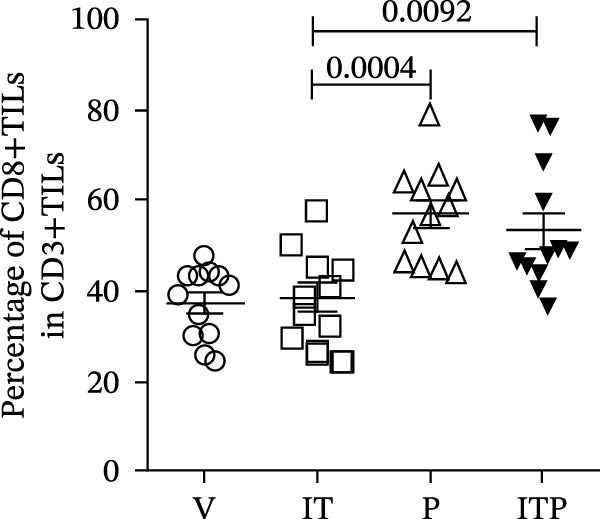
(C)

**Figure:**
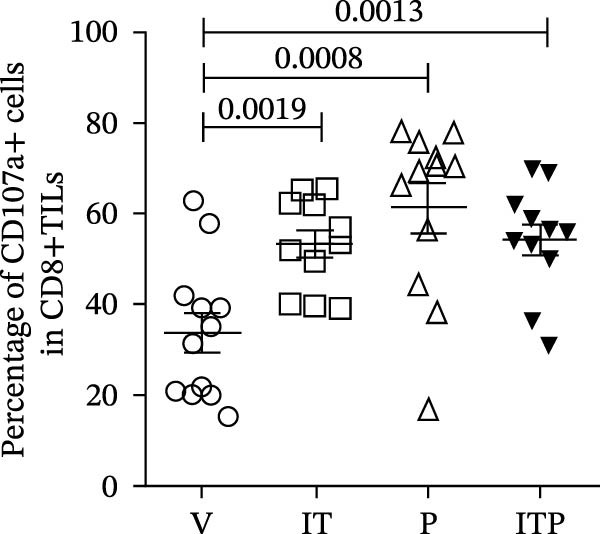
(D)

Figure 4 shows the cytokine production profile of CD8+TILs after stimulated single cell suspension of the tumor tissues with PMA/ionomycin for 4 h ex vivo. Flow cytometry gating strategies are shown in Figure S2. Production of IFN‐γ was similar among all the groups (Figure 4A,B). When compared with the V group, other groups had significantly greater number of TNF‐α producer (Figure 4A,C). In the PD1 blockade and combined treatment groups, the proportion of TNF‐α and IL‐2 producers was significantly greater than the IT group, especially in the combined treatment group, which contributed the largest number of IL‐2 producers (Figure 4A,D).

Figure 4Cytokine production of CD8+TILs (*n* = 5 in each group). (A) Gating on CD8+TILs, contour plots showed the expression of IFN‐γ, TNF‐α, and IL‐2 of different treatment groups. (B–D) The ratio of IFN‐γ+ cells (B), TNF‐α+ cells (C), and IL‐2+ cells (D) in CD8+TILs. Differences among different treatment groups were evaluated using an unpaired Student’s *t*‐test. The experimental data were confirmed through replication in three independent experiments. IT, intermittent tacrolimus treatment group; ITP, combined treatment group (intermittent tacrolimus + PD1 blockade); P, PD1 blockade group; TILs, tumor‐infiltrating lymphocytes; V, vehicle group.

**Figure:**
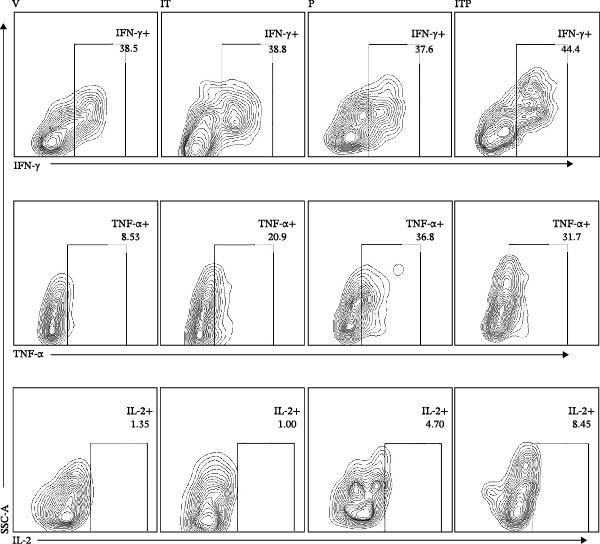
(A)

**Figure:**
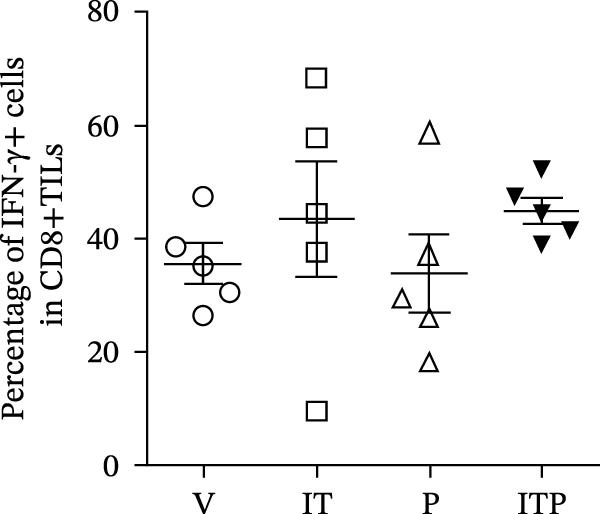
(B)

**Figure:**
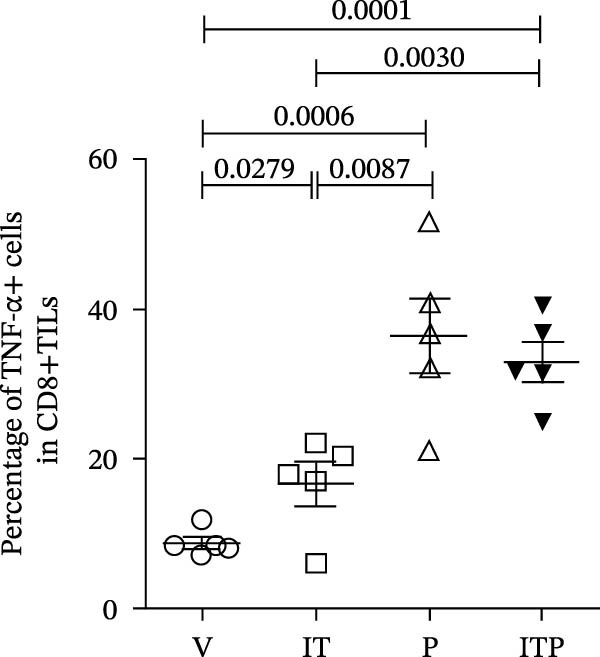
(C)

**Figure:**
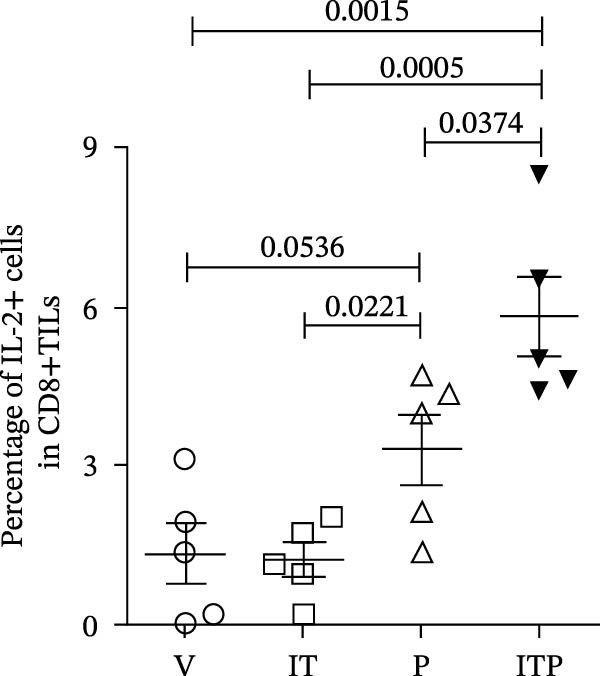
(D)

### 3.3. Tacrolimus Reshaped the Exhaustion Phenotype of CD8+TILs

The expression of IRs on CD8+TILs was analyzed. Initially, the authors observed a significant increase in the expression of PD1 and CD223 in CD8+TILs from IT and/or PD1 blockade treatment groups (Figure 5A–C). Furthermore, the IT group exhibited a significantly lower CD223 expression compared to the PD1 blockade group (Figure 5B). Subsequently, the authors conducted a detailed analysis of the PD1hi (PD1+CD223+) and PD1int (PD1+CD223−) populations in CD8+TILs.

Figure 5The expression of PD1 and CD223 in CD8+TILs (*n* = 11 or 12 in each group). (A) Contour plots showed the expression of PD1 and CD223 in CD8+TILs of different treatment groups. Upper panel: gating strategy based on PD1‐FMO and CD223‐FMO; lower panel: the expression of PD1 and CD223 in CD8+TILs of different treatment groups. (B, C) The ratio of CD223+ or PD1+cells in CD8+TILs. (D) The ratio of PD1hi CD8+TILs. (E) The ratio of PD1int CD8+TILs. Differences among different treatment groups were evaluated using an unpaired Student’s *t*‐test. The experimental data were confirmed through replication in three independent experiments. IT, intermittent tacrolimus treatment group; ITP, combined treatment group (intermittent tacrolimus + PD1 blockade); P, PD1 blockade group; PD1hi, high PD1 expressing subset (PD1+CD223+); PD1int, intermediate PD1 expressing subset (PD1+CD223‐); TILs, tumor‐infiltrating lymphocytes; V, vehicle group.

**Figure:**
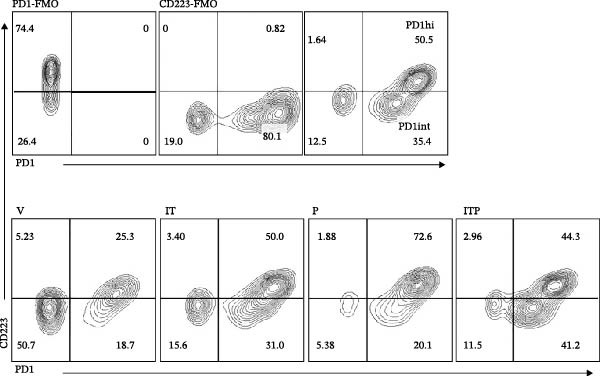
(A)

**Figure:**
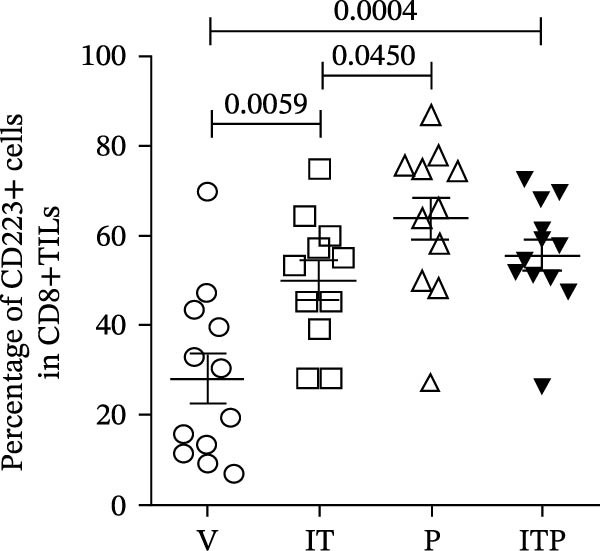
(B)

**Figure:**
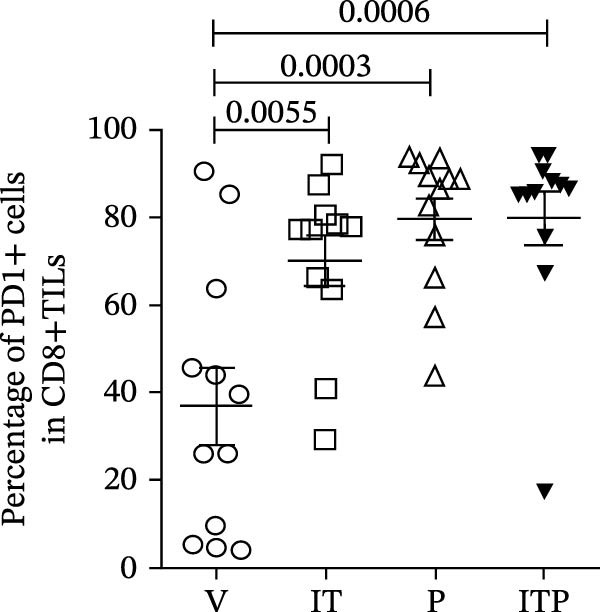
(C)

**Figure:**
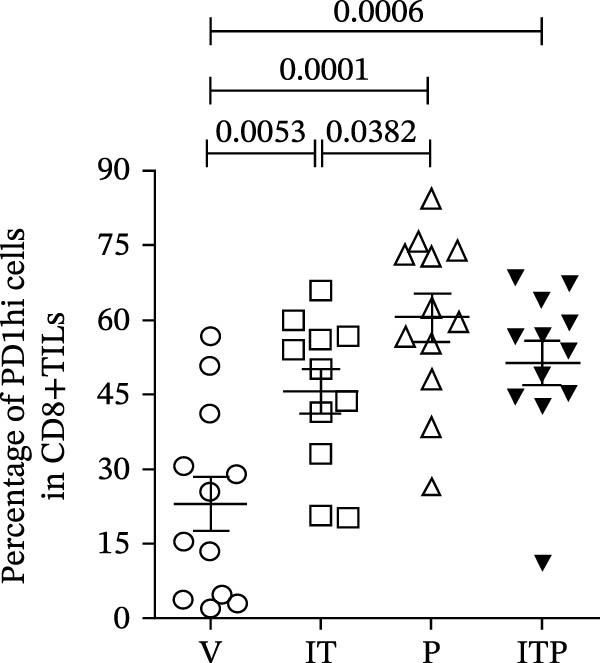
(D)

**Figure:**
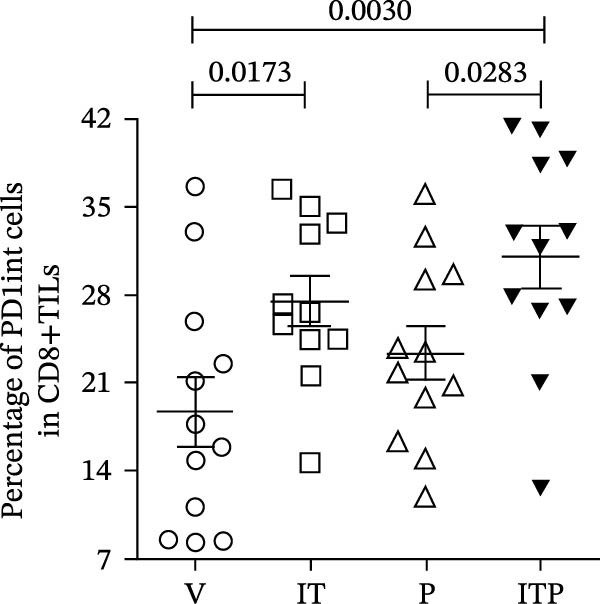
(E)

When compared to the V group, tacrolimus treatment (IT group or combined treatment group) significantly elevated the numbers of both PD1hi and PD1int CD8+TILs, but PD1 blockade only increased the population of PD1hi CD8+TILs (Figure 5D,E).

In comparison to the PD1 blockade group, the combined treatment group significantly increased the number of PD1int CD8+TILs but not PD1hi CD8+TILs (Figure 5D,E). Although IT group had similar proportion of PD1int CD8+TILs with PD1 blockade, the proportion of PD1hi CD8+TILs in IT group was significantly lower (Figure 5D,E). These results indicated that compared to PD1 blockade, tacrolimus reshaped the exhaustion phenotype of CD8+TILs, restoring a balance between PD1int and PD1hi populations.

### 3.4. Combined Treatment Increased the Progenitor Population of CD8+TILs

TCF1 was necessary to generate the stem‐like progenitor CD8+TILs. In a murine B16 melanoma model, intertumoral TCF1+PD1+CD8+TILs expressed markers of stem cells [21]. As Ly108 was a surrogate for TCF1, we detected Ly108+PD1+ cells in PD1int or PD1hi populations of CD8+TILs 6 days post IT administration. As shown in Figure 6A,B, combined treatment contributed significantly higher numbers of Ly108+ cells in both PD1int and PD1hi populations. Numbers of CD127+ cells in PD1int and PD1hi populations after treatment followed a similar trend as that of Ly108+ cells (Figure 7).

Figure 6Combined treatment increased the expression of Ly108 in CD8+TILs (*n* = 5 in each group). (A) Gate on CD8+TILs: the contour plot showed the expression of Ly108 in PD1hi CD8+TILs and PD1int CD8+TILs. (B) Statistical analysis of the ratio of Ly108+cells in PD1hi or PD1int CD8+TILs. Differences among different treatment groups were evaluated using an unpaired Student’s *t*‐test. The experimental data were confirmed through replication in three independent experiments. IT, intermittent tacrolimus treatment group; ITP, combined treatment group (intermittent tacrolimus + PD1 blockade); P, PD1 blockade group; PD1hi, high PD1 expressing subset (PD1+CD223+); PD1int, intermediate PD1 expressing subset (PD1+CD223−); TILs, tumor‐infiltrating lymphocytes; V, vehicle group.

**Figure:**
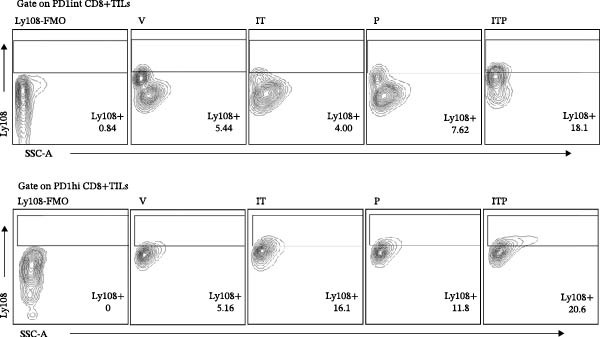
(A)

**Figure:**
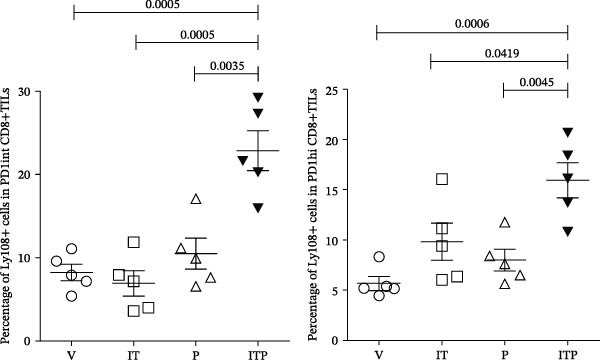
(B)

Figure 7Combined treatment increased the expression of CD127 in CD8+TILs (*n* = 5 in each group). (A) Gate on CD8+TILs, contour plot showed the expression of CD127 in PD1hi CD8+TILs and PD1int CD8+TILs. (B) Statistical analysis of the ratio of CD127+cells in PD1hi or PD1int CD8+TILs. Differences among different treatment groups were evaluated using an unpaired Student’s *t*‐test. The experimental data were confirmed through replication in three independent experiments. IT, intermittent tacrolimus treatment group; ITP, combined treatment group (intermittent tacrolimus + PD1 blockade); P, PD1 blockade group; PD1hi, high PD1 expressing subset (PD1+CD223+); PD1int, intermediate PD1 expressing subset (PD1+CD223−); TILs, tumor‐infiltrating lymphocytes; V, vehicle group.

**Figure:**
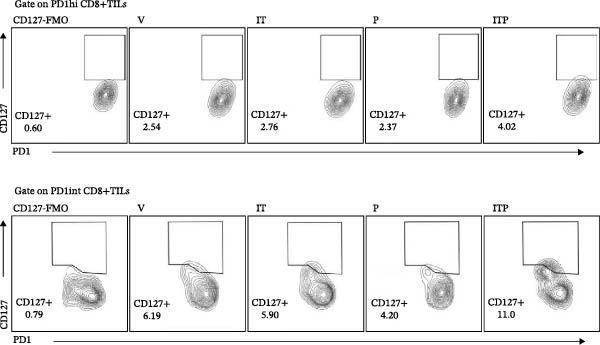
(A)

**Figure:**
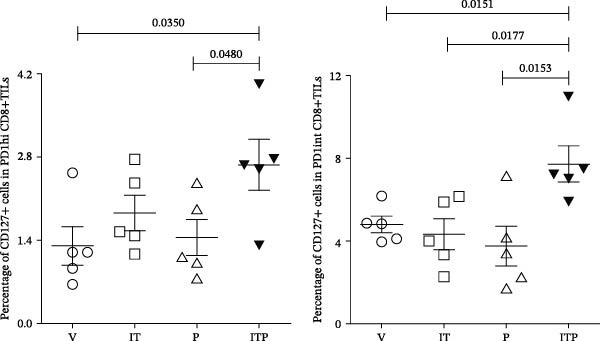
(B)

### 3.5. IT Potentiated the Antitumor Effects of PD1 Blockade in Melanoma‐Bearing Mice

The assessment of tumor growth following IT and/or PD1 blockade treatment was conducted. Tumor‐bearing mice received tacrolimus every 5 days, with PD1 blocking antibody administered 1 day before tacrolimus, as outlined in the treatment schedule depicted in Figure 8A. Notably, the combined treatment of IT and PD1 blockade significantly curtailed the tumor growth rate (Figure 8B). Both IT and PD1 blockade individually contributed to a significant reduction in tumor weight 15 days after the onset of treatment, with the combined treatment group displaying the lowest average tumor weight among the groups (Figure 8C,D). Fifteen days posttreatment onset, mouse body weights were similar among different groups (Table S1 and Figure S3).

Figure 8Effects of intermittent tacrolimus and/or PD1 blockade on melanoma growth (*n* = 10 in each group). (A) Experimental design. (B) Average B16F10 tumor volumes were represented as the mean ± SEM. (C) Tumor weight on Day 15 was represented as the mean ± SEM. (D) Excised tumors on Day 15. Tumor growth curves were analyzed by repeated‐measures two‐way ANOVA with Greenhouse–Geisser correction and Tukey’s post hoc test. Differences of tumor weight among different treatment groups were evaluated using an unpaired Student’s *t*‐test. The experimental data were confirmed through replication in four independent experiments. IT, intermittent tacrolimus treatment group; ITP, combined treatment group (intermittent tacrolimus + PD1 blockade); P, PD1 blockade group; V, vehicle group.

**Figure:**
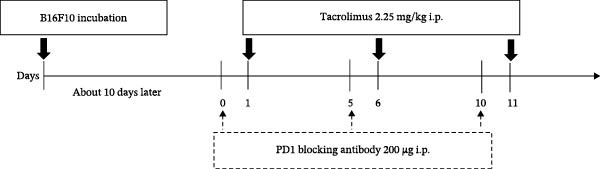
(A)

**Figure:**
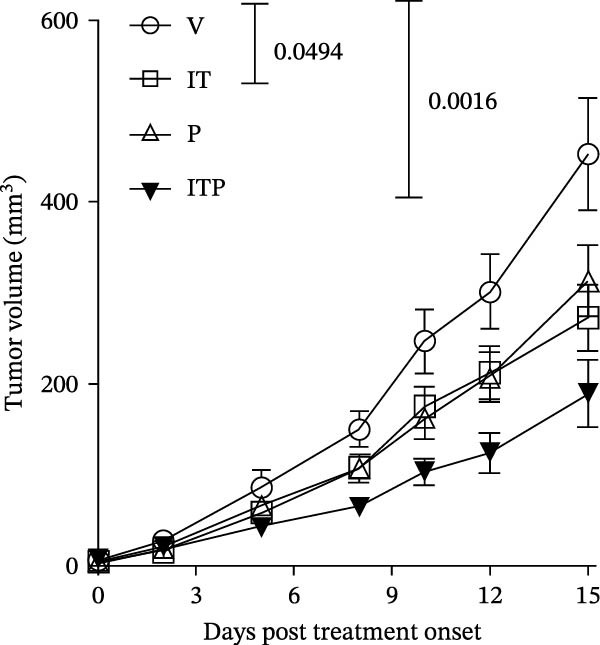
(B)

**Figure:**
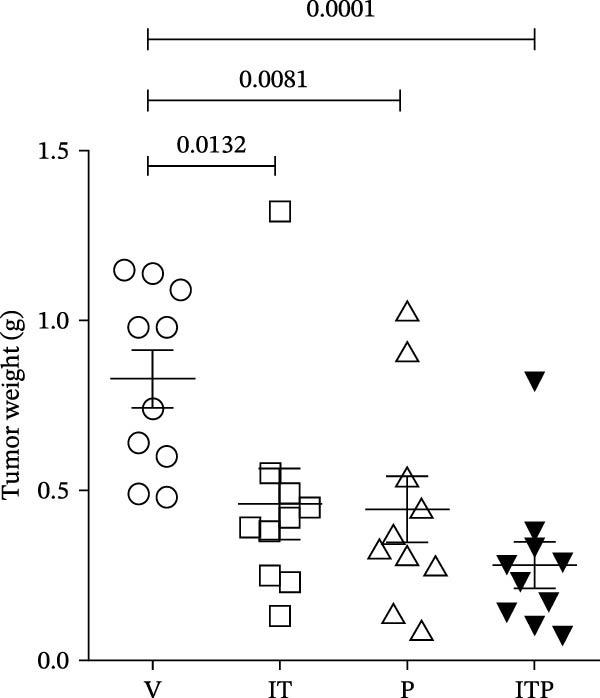
(C)

**Figure:**
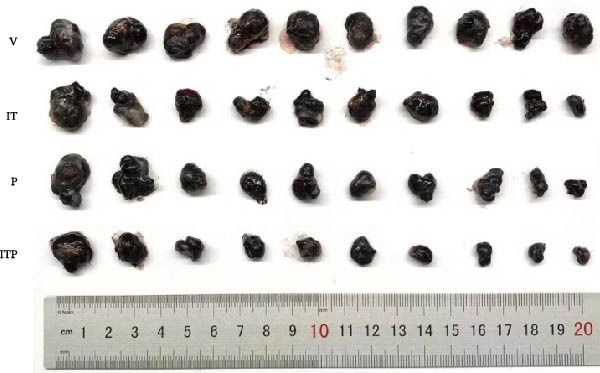
(D)

At last, tumor specific CD8+TILs were detected by stimulating tumor cell suspension with Trp2_180–188_ peptide. Before peptide stimulation, some IFN‐γ or TNF‐α producing CD8+TILs already existed in tumor cell suspension, around 39% producing IFN‐γ and 10% producing TNF‐α (Figure 9A). After incubating tumor cell suspension with Trp2_180–188_ peptide for 4 h, the number of IFN‐γ or TNF‐α producers in the IT group further increased, which was significantly higher than the V group. The average percentage of TNF‐α producers was 12% in the IT group and 9% in the V group. The average percentage of IFN‐γ producers increased from 34% in the V group to 43% in the IT group (Figure 9A–C). The results indicated an enhanced CD8+ T cell responses in the IT group, specifically against melanoma Trp2 antigen.

Figure 9Cytokine production of CD8+TILs after Trp2_180–188_ peptide stimulation (*n* = 6 in each group). (A) Gating on CD8+TILs, contour plots showed the expression of IFN‐γ and TNF‐α of different treatment groups. Left pannel: positive control (PMA/Ion) and negative control (no stimulaton). Right pannel: tumor tissue suspension from vehicle and IT groups were stimulated with Trp2_180–188_ peptide. (B, C) Statistical analysis of the ratio of IFN‐γ+ cells (B) and TNF‐α+ cells (C) in CD8+TILs. PMA/Ion: Tumor tissue suspension was stimulated with PMA and ionomycin. Differences among different treatment groups were evaluated using an unpaired Student’s *t*‐test. The experimental data were confirmed through replication in three independent experiments. IT, intermittent tacrolimus treatment group; TILs, tumor‐infiltrating lymphocytes; V, vehicle group.

**Figure:**
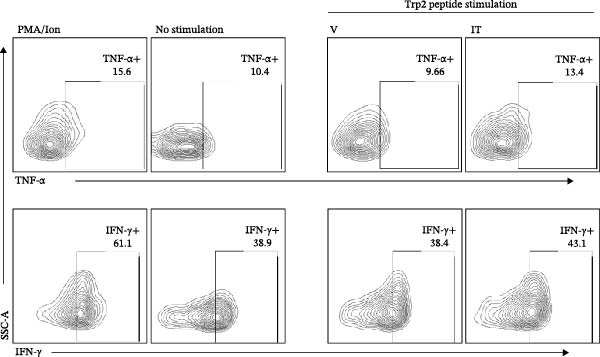
(A)

**Figure:**
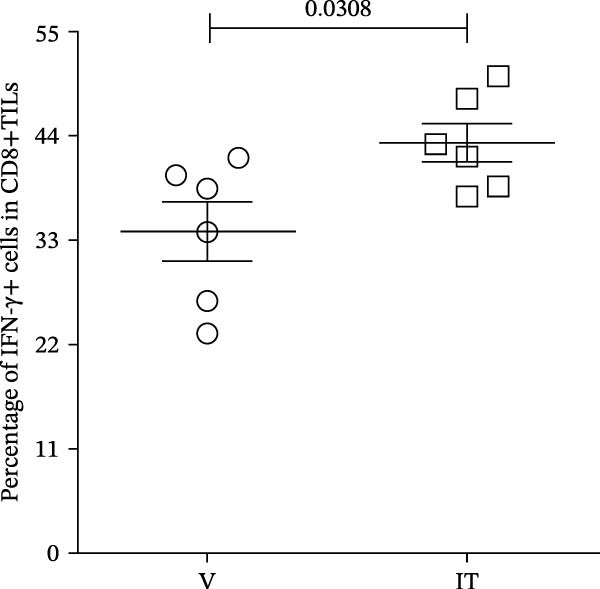
(B)

**Figure:**
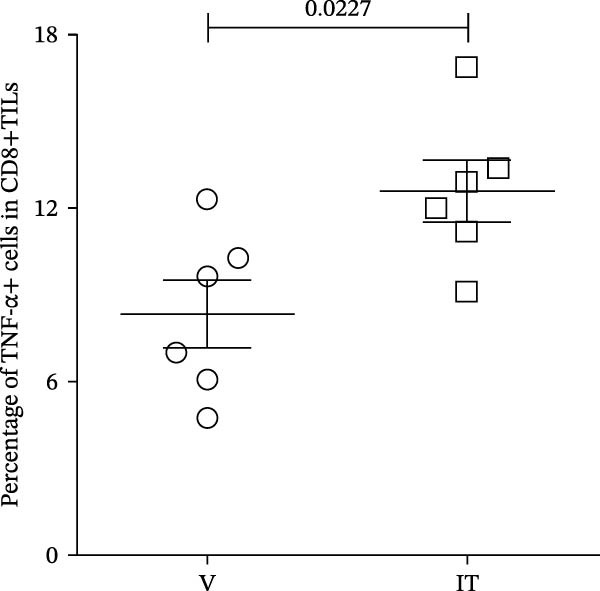
(C)

## 4. Discussion

In the context of acute infection and vaccination, the majority of effector CD8+ T cells undergo apoptosis following the clearance of antigens. However, a small subset of these cells can transition into a quiescent memory T cell population, providing long‐lasting immunity. Conversely, in the presence of chronic infection and cancer, where antigenic stimulation persists, the effector CD8+ T cells deviate from the pathway of memory differentiation and embark on an alternative trajectory leading to exhaustion. This subset of CD8+ T cells sustains upregulation of multiple IRs and alters expression and use of key transcription factors and metabolic derangements, leading to clearance of antigen‐specific CD8+T cells [22–24].

The genesis of exhausted CD8+T cells involves an intricate interplay of signals originating from the TCR, co‐IRs, and cytokines. The precise mechanisms through which these signals crosstalk and contribute to the developmental transition of exhaustion remain uncertain. Notably, there is unanimous acknowledgment that TCR signaling plays a pivotal role in the evolution of exhaustion. During the early stages, the exhaustion state is reversible. T cells isolated from mice within the first week of chronic infection demonstrated the capacity to form memory T cells upon adoptive transfer into noninfected mice. However, this ability diminished significantly when T cells were isolated at 2 and 4 weeks postinfection [25]. In human cells, purified CD8+T cells underwent continuous in vitro stimulation, leading to T cell exhaustion. Notably, the exhaustion phenotype exhibited partial reversibility when TCR stimuli were removed at the early stage of stimulation [1].

These pieces of evidence substantiate the notion that TCR stimulation signals stand as one of the primary drivers for exhaustion differentiation. Furthermore, TCR activation signals have been identified as the principal force behind the upregulation of IRs. The expression of PD1 on virus‐specific CD8+ T cells undergoes rapid upregulation within 24 h upon infection, preceding any cell division. This swift PD1 expression is not observed when the host is infected with a virus strain featuring a mutation in the CD8+ T cell epitope [26]. Our previous work also demonstrated that functional tumor specific CD8+ T cells in spleen expressed a high level of PD1 [27]. The induction of other IRs, including CD223 and TIM3, was similarly prompted by TCR signals. Within the tumor microenvironment, CD8+TILs specific to the tumor expressed elevated levels of PD1, CD223, and TIM3 in comparison to non‐tumor specific CD8+TILs [7, 28]. Given the robust correlation between T cell activation signals and the expression of multiple IRs, numerous researchers have regarded co‐IRs, particularly PD1, as markers for identifying activated T cells [29, 30]. This perspective was further validated in patients with non‐small cell lung cancer. TILs not only co‐expressed PD1 with other IRs such as TIM3 and CD223 but also exhibited a recently activated, non‐exhausted phenotype [28]. Therefore, in the current study, memory markers (such as Ly108 and CD127) were also applied collectively to distinguish intermediate and terminally exhausted subsets of CD8+TILs.

The degree of exhaustion was found to be closely linked to the antigen load and the duration of stimulation. The effective activation of naïve CD8+T cells required ~15–20 h in response to high doses of antigen and co‐stimulation. In effector CD8+ T cells, continuous exposure to antigen for more than 12 h led to nearly complete inhibition of proliferation [31, 32]. Furthermore, a higher antigen load was observed to correlate with a more pronounced exhaustion of CD8+ T cells [12, 33, 34]. Based on the strong correlation between antigen stimulation and the extent of exhaustion, this study viewed exhaustion as an over‐activation status induced by prolonged antigen stimulation.

We found that a greater proportion of PD1hi population than PD1int population produced IFN‐γ or TNF‐α after in vitro stimulation. These findings were consistent with previous reports, which showed that terminally exhausted CD8+ T cells contributed the largest number of IFN‐γ and TNF‐α producers, while both the intermediately and terminally exhausted populations mediated the most target killing [9]. Another study demonstrated that terminally exhausted CD8+ T cells (Ly108‐Tim3+) expressed more granzyme B than did progenitor exhausted cells (Ly108+Tim3−) and were more cytotoxic in direct cytotoxicity assays with tumor‐cell targets, but in vivo tumor control was significantly greater in animals that received progenitor exhausted cells, probably due to their improved survival and ability to differentiate into cytotoxic terminally exhausted CD8+ T cells [10].

Molecules that conduct signals downstream of the TCR activation pathway play a pivotal role in inducing exhaustion. Previous studies have demonstrated that transcription factors responsible for initiating T‐cell activation are also implicated in anergy and exhaustion, such as the NFAT family, NR4A family, TOX, Eomes, Blimp‐1, Batf, HIF‐1, FoxO1, and FoxP1, among others [7, 35–38]. However, as of now, no transcription factors specifically designated as “exhaustion‐specific” have been identified. Given that exhaustion is induced by robust or chronic antigen stimulation, researchers have employed tacrolimus (an activation inhibitor) to modulate continuous antigen stimulation signals, thereby aiming to restrain the exhaustion process in CD8+ T cells.

This study uncovered that IT and/or PD1 blockade facilitated the infiltration of functional CD8+TILs. In the PD1 blockade group, there was a notable increase in the proportion of PD1hi CD8+TILs, while the proportion of PD1int CD8+TILs resembled that of the V group. PD1 blockade led to a reshaping of the CD8+T cell exhaustion phenotype, displaying markedly heightened expression levels of multiple IRs. Conversely, IT treatment showed a lower proportion of PD1hi CD8+TILs. This suggests that IT treatment restored a balance between PD1int and PD1hi populations, indicating a constrained exhaustion profile in the IT‐treated cohorts.

This study aimed to demonstrate the potential of IT treatment in constraining the developmental process of CD8+TILs exhaustion. Nevertheless, similar to any research endeavor, the authors acknowledge its inherent limitations. First, the study was conducted using a singular melanoma tumor model, which limits the generalizability of the findings to other tumor types. Second, the mechanism of IT therapy to enhance the antitumor immunity remains unclear, which needs to be investigated in future research. Although this research delved into the exhaustion phenotype of PD1+CD8+TILs, the evaluation would be more precise if conducted on tumor‐specific CD8+TILs, given that PD1 identifies the patient‐specific CD8+ tumor‐reactive repertoire infiltrating human tumors [39, 40].

## 5. Conclusions

This study compared PD1 blockade therapy with IT treatment in melanoma‐bearing mice and revealed that both treatment strategies increased the quantity of functional CD8+TILs. The IT treatment delayed the development of exhaustion in CD8+TILs, manifested by a higher proportion of CD8+TILs with moderate IRs expression, whereas the PD1 blockade group exhibited a much higher proportion of CD8+TILs with elevated IRs expression.

## Author Contributions

Zili Wang conceptualized and Junjin Lin designed this study. Darong Chen, Sisi Feng, Zhaolong Chen, Zhisheng Weng, and Shu Zhang performed experiments. Darong Chen and Sisi Feng analyzed the data in this article. Zili Wang wrote the manuscript. Junjin Lin reviewed and supervised the experiments.

## Funding

This research was supported by the Natural Science Foundation of Fujian Province (Grant 2022J01223), Startup Fund for Scientific Research of Fujian Medical University (Grant 2022QH1089), the Natural Science Foundation of Fujian Province (Grant 2024J01570), and the Natural Science Foundation of Fujian Province (Grant 2023J01314).

## Disclosure

All authors agree to be accountable for all aspects of work ensuring integrity and accuracy.

## Ethics Statement

All of the experimental protocols were approved by Fujian Medical University, Faculty of Animal Care, Housing, and Procedures. This study adhered strictly to IACUC guidelines and policies, maintaining the highest level of ethical standards from its pre‐implementation, implementation, and post‐implementation. The materials, methods, and procedures used in this study undergone rigorous scrutiny and approval by Fujian Medical University.

## Conflicts of Interest

The authors declare no conflicts of Interest.

## Supporting Information

Additional supporting information can be found online in the Supporting Information section.

## Supporting information


**Supporting Information** This manuscript contains three Supporting Information figures and one Supporting Information table. Supporting Information Figures S1 and S2 described the flow cytometry gating strategy for the exhaustion markers and intracellular cytokines, respectively. Supporting Information Figure S3 described mouse body weights 15 days post treatment onset. Supporting Information Table S1 described numerical values of mouse body weights 15 days post treatment onset.

## Data Availability

All data generated or analyzed during this study are included in this published article. All raw data are available upon request from the corresponding author.
