# Supplementary material for: Intermittent Tacrolimus Treatment Delays CD8+ Tumor‐Infiltrating Lymphocyte Exhaustion and Enhances PD1 Blockade Therapy in Melanoma‐Bearing Mice
Source: J Immunol Res. 2026 Feb 16;2026:8444562. doi: 10.1155/jimr/8444562 (PMC13140372; doi:10.1155/jimr/8444562)
Supplement: Supplementary file 1 — Supporting Information This manuscript contains three Supporting Information figures and one Supporting Information table. Supporting Information Figures S1 and S2 described the flow cytometry gating strategy for the exhaustion markers and intracellular cytokines, respectively. Supporting Information Figure S3 described mouse body weights 15 days post treatment onset. Supporting Information Table S1 described numerical values of mouse body weights 15 days post treatment onset. [file JIMR-2026-8444562-s001.doc]

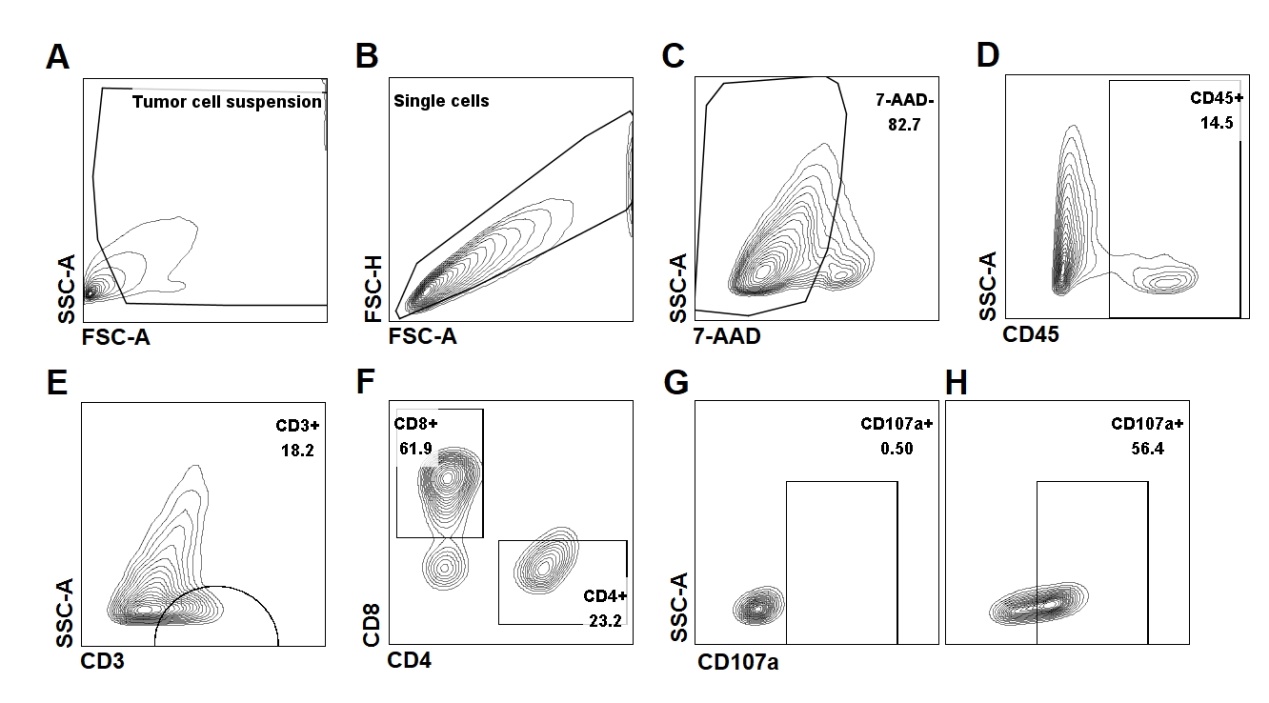


**Supplementary Figure 1**

**Supplementary Figure 1.** Flow cytometry gating strategy. (A) Contour plots showed all cell population in the tumor tissue cell suspension. (B) Contour plots showed the single cell population in the overall population from A. (C) Contour plots showed the viable cells (7-AAD negative) in the single cell population from B. (D) Contour plots showed the CD45+ immune cells in the viable cells from C. (E) Contour plots showed the CD3+TILs in the CD45+ immune cells from D. (F) Contour plots showed the CD8+ or CD4+ TILs in the CD3+TILs from E. (G) Gating on CD8+TILs, contour plots showed the CD8+TILs stained without CD107a-BV421 (CD107a-FMO). (H) Gating on CD8+TILs, contour plots showed the CD8+TILs stained with CD107a-BV421. TILs, tumor-infiltrating lymphocytes.


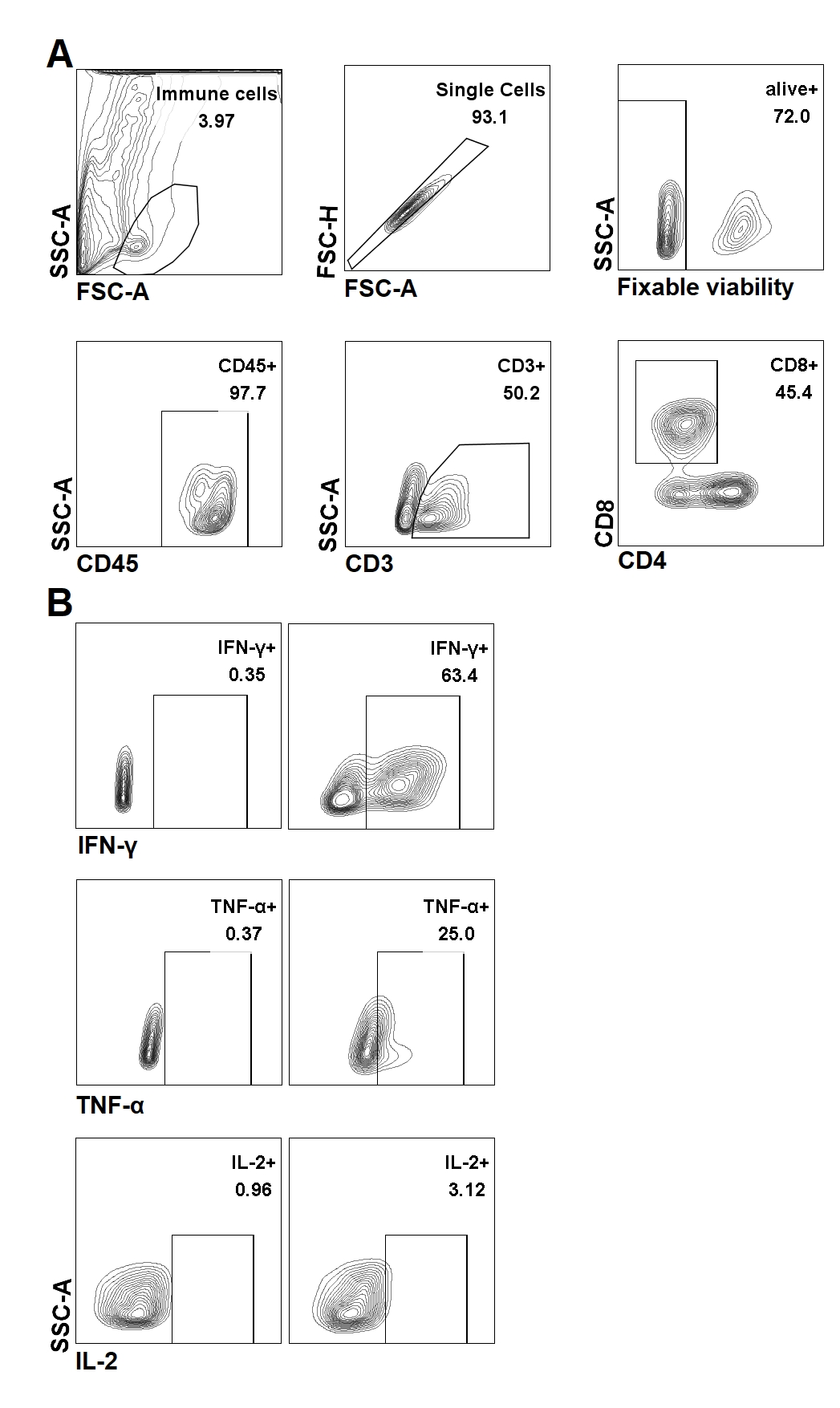


**Supplementary Figure 2**

**Supplementary Figure 2.** Flow cytometry gating strategy for intracellular cytokine staining. Single cell suspension of melanoma tissue was co-cultured with PMA/ionomycin for 4h ex vivo. (A) Contour plots showed CD8+TILs. (B) Contour plots showed the CD8+TILs stained with or without IFN-γ-PE, TNF-α-BV421 and IL-2-PE-Cy7 (IFN-γ-FMO, TNF-α-FMO, IL-2-FMO).

**Supplementary Table 1**

| **Supplementary Table 1**  Mouse body weights (g) in different treatment groups 15 days post treatment onset | | | | |
| --- | --- | --- | --- | --- |
| Groups | V | IT | P | ITP |
|  | 20.4 | 19.0 | 20.2 | 18.1 |
|  | 17.6 | 19.3 | 19.6 | 18.2 |
|  | 19.4 | 17.7 | 19.1 | 18.5 |
|  | 18.5 | 19.5 | 17.3 | 18.6 |
|  | 18.5 | 19.4 | 18.6 | 19.1 |
|  | 19.2 | 19.0 | 18.7 | 19.4 |
|  | 18.0 | 21.2 | 18.9 | 18.3 |
|  | 19.3 | 20.9 | 18.7 | 18.4 |
|  | 17.3 | 18.6 | 18.5 | 18.7 |
|  | 21.4 | 17.3 | 17.9 | 18.2 |

**Supplementary Figure 3**


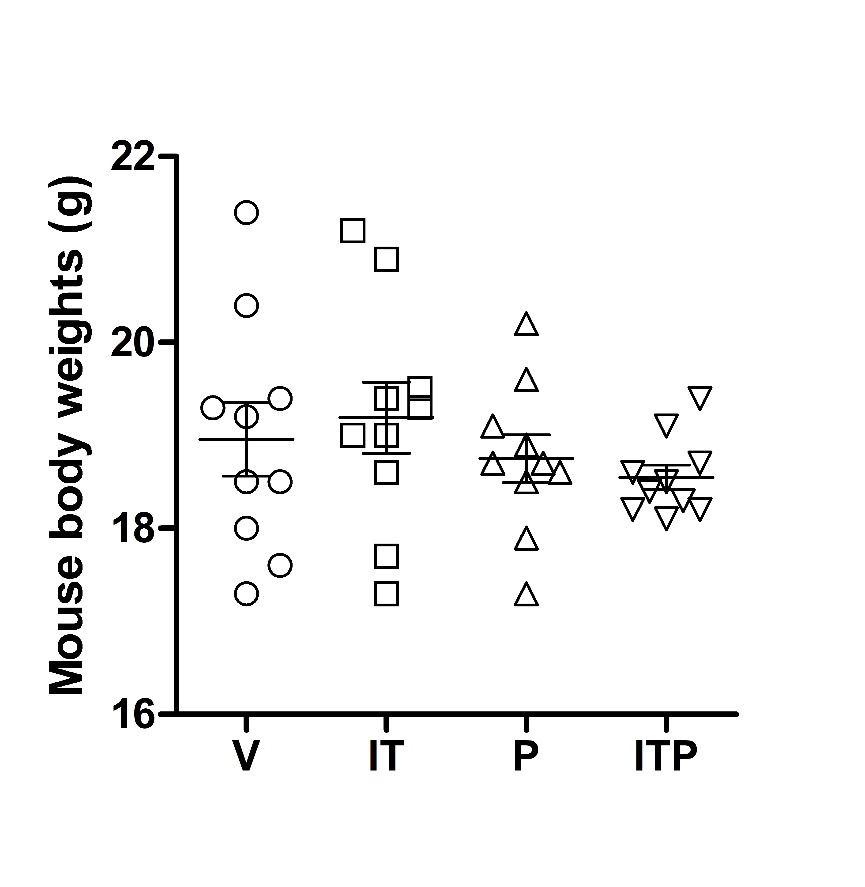


**Supplementary Figure 3.** Mouse body weights measured 15 days post treatment onset (mean ± SEM). V, Vehicle group; IT, intermittent tacrolimus treatment group; P, PD1 blockade group; ITP, Combined treatment group (intermittent tacrolimus + PD1 blockade).
